# Co-Designing a Consumer-Focused Digital Reporting Health Platform to Improve Adverse Medicine Event Reporting: Protocol for a Multimethod Research Project (the ReMedi Project)

**DOI:** 10.2196/60084

**Published:** 2025-01-15

**Authors:** Eyob Alemayehu Gebreyohannes, Christopher Thornton, Myra Thiessen, Sieta T de Vries, Andre Q Andrade, Lisa Kalisch Ellett, Oliver Frank, Phaik Yeong Cheah, Kim-Kwang Raymond Choo, Tracey Lea Laba, Elizabeth E Roughead, Indae Hwang, Geraldine Moses, Renly Lim

**Affiliations:** 1 Quality Use of Medicines and Pharmacy Research Centre UniSA Clinical and Health Sciences University of South Australia Adelaide Australia; 2 School of Allied Health The University of Western Australia Perth Australia; 3 UniSA Creative University of South Australia Adelaide Australia; 4 Monash Art, Design and Architecture Monash University Melbourne Australia; 5 Department of Clinical Pharmacy and Pharmacology University of Groningen University Medical Center Groningen Groningen Netherlands; 6 UniSA Clinical and Health Sciences University of South Australia Adelaide Australia; 7 Discipline of General Practice, Adelaide Medical School Faculty of Health and Medical Sciences The University of Adelaide Adelaide Australia; 8 Oakden Medical Centre Adelaide Australia; 9 Centre for Tropical Medicine and Global Health Nuffield Department of Medicine University of Oxford Oxford United Kingdom; 10 Mahidol Oxford Tropical Medicine Research Unit Faculty of Tropical Medicine Mahidol University Bangkok Thailand; 11 The Ethox Centre Nuffield Department of Population Health University of Oxford Oxford United Kingdom; 12 Department of Information Systems and Cyber Security The University of Texas at San Antonio San Antonio, TX United States; 13 Centre for Health Economics, Research and Evaluation University of Technology Sydney Sydney Australia; 14 School of Pharmacy and Pharmaceutical Sciences University of Queensland Brisbane Australia

**Keywords:** adverse drug events, drug-related side effects and adverse reactions, adverse drug reaction reporting systems, pharmacovigilance, digital health, medication safety, co-design, qualitative research, user-centered design

## Abstract

**Background:**

Adverse medicine events (AMEs) are unintended effects that occur following administration of medicines. Up to 70% of AMEs are not reported to, and hence remain undetected by, health care professionals and only 6% of AMEs are reported to regulators. Increased reporting by consumers, health care professionals, and pharmaceutical companies to medicine regulatory authorities is needed to increase the safety of medicines.

**Objective:**

We describe a project that aims to co-design a digital reporting platform to improve detection and management of AMEs by consumers and health care professionals and improve reporting to regulators.

**Methods:**

The project will be conducted in 3 phases and uses a co-design methodology that prioritizes equity in designing with stakeholders. Our project is guided by the Consolidated Framework for Implementation Research. In phase 1, we will engage with 3 stakeholder groups—consumers, health care professionals, and regulators—to define digital platform development standards. We will conduct a series of individual interviews, focus group discussions, and co-design workshops with the stakeholder groups. In phase 2, we will work with a software developer and user interaction design experts to prototype, test, and develop the digital reporting platform based on findings from phase 1. In phase 3, we will implement and trial the digital reporting platform in South Australia through general practices and pharmacies. Consumers who have recently started using medicines new to them will be recruited to use the digital reporting platform to report any apparent, suspected, or possible AMEs since starting the new medicine. Process and outcome evaluations will be conducted to assess the implementation process and to determine whether the new platform has increased AME detection and reporting.

**Results:**

This project, initiated in 2023, will run until 2026. Phase 1 will result in persona profiles and user journey maps that define the standards for the user-friendly platform and interactive data visualization tool or dashboard that will be developed and further improved in phase 2. Finally, phase 3 will provide insights of the implemented platform regarding its impact on AME detection, management, and reporting. Findings will be published progressively as we complete the different phases of the project.

**Conclusions:**

This project adopts a co-design methodology to develop a new digital reporting platform for AME detection and reporting, considering the perspectives and lived experience of stakeholders and addressing their requirements throughout the entire process. The overarching goal of the project is to leverage the potential of both consumers and technology to address the existing challenges of underdetection and underreporting of AMEs to health care professionals and regulators. The project potentially will improve individual patient safety and generate new data for regulatory purposes related to medicine safety and effectiveness.

**International Registered Report Identifier (IRRID):**

DERR1-10.2196/60084

## Introduction

Adverse medicine events (AMEs), also known as adverse drug events, are unintended effects that occur following administration of a medicine and include adverse reactions and harm from medication errors [1]. AMEs are common and result in patient harm. In Australia, an estimated 1.2 million people reportedly experienced an AME within a 6-month period [2]. While AMEs can occur in anyone, people with chronic conditions and older people are particularly vulnerable to and are most affected by AMEs. For example, 1 in 5 hospital admissions of older adults in Australia is due to AME [2-4]. According to a 2022 estimate, medicine-related hospital admissions, including instances of noncompliance, overdose, and AME, incur an estimated annual cost of Aus $1.4 billion (US $870 million) in Australia, with AME being the most prevalent contributing factor [2,5]. Early detection and management of AMEs are crucial to preventing avoidable harms such as medicine-related falls, hospitalizations, and deaths. However, findings from surveys and reviews of consumer medical records conducted internationally suggest that many consumers do not disclose their AMEs unless prompted to do so. Consequently, up to 70% of AMEs are undetected by health care professionals [6,7], emphasizing the need for proactive interventions to identify and resolve AMEs.

AMEs are also underreported to medicine regulatory authorities (“regulators”), making it difficult to understand how medicines affect consumers. Spontaneous reporting of AMEs is the most common mechanism of safety surveillance worldwide after a medicine has been introduced to the market [8]. Spontaneous reporting of AMEs by consumers, health care professionals, and pharmaceutical companies is vital for regulators to identify potential medicine safety signals [9] and—when relevant—mandate necessary changes, such as updating product labels or withdrawing medicines from the market. A major challenge, however, is the very low AME reporting rate; as evidenced by a systematic review of 37 studies from 12 countries, only an estimated 6% of AMEs experienced by patients were reported [10].

Consumers often detect AMEs before their health care professionals notice them [11], and, where patient engagement is implemented, consumer self-report of AMEs alerts regulators to new and previously unknown reactions prior to health professional reports [12,13]. In Australia, however, the number of reports to regulators from consumers is disproportionately low compared with those made by health care professionals and pharmaceutical manufacturers [14], partly because of consumers’ limited awareness of the reporting system and perceived absence of benefits of reporting [15]. The AME reporting system, developed by the medicine regulatory body, the therapeutic goods administration (TGA) [14], has seen limited consumer uptake in Australia. Furthermore, there is currently no Australian-specific AME reporting platform co-designed with consumers. To address this gap, we developed a prototype system in a small pilot project comprising both Android and iOS apps and a public-facing website for consumers to report any AMEs they experienced [16]. The system was shown to be user-friendly. However, the development involved limited stakeholder engagement and participation (3 consumers) due to the inherent nature of a small pilot project.

Building on this pilot project, the current project aims to co-design with stakeholders (consumers, health care professionals, and regulators) a digital reporting platform to improve AME detection, management, and reporting. The ultimate goal of this project is to empower consumers to actively detect, manage, and report AMEs, fostering a collaborative approach with their health care professionals, and at the same time improve AME reporting to the TGA.

## Methods

### Study Design

**Figure 1:**
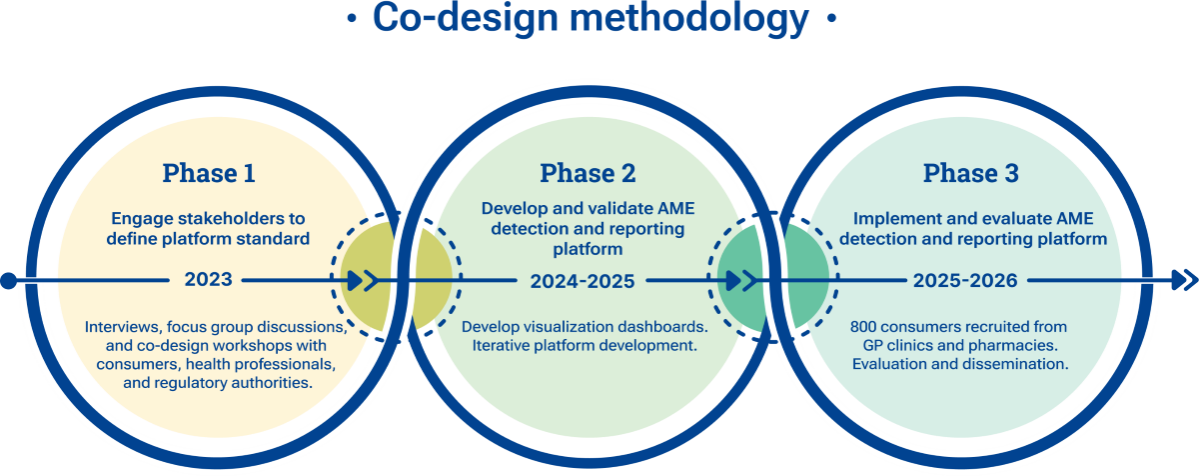
Key activities in each phase of the project. AME: adverse medicine event; GP: general practitioner.

The project is being conducted in 3 phases (Figure 1) from 2023 to 2026 and uses a co-design methodology that prioritizes designing equitably with all stakeholders [17]. Co-design methodology focuses on generating and reflecting on data related to people’s lived experiences and engaging participants in action to enhance the quality of their lives. Co-design facilitates collaboration among stakeholders to address challenges within sociotechnical systems and daily services [18,19]. It uses design-based strategies to collect qualitative data around users’ experience and the interests of the stakeholders providing services to those users to foster dialogue for mutual insights. Using a range of research methods including stakeholder workshops, focus group discussions, and interviews, co-design aims to describe, categorize, question, and evaluate the needs, experiences, opinions, interests, decisions, and behaviors of stakeholders, ensuring equity through structured reflection. This project builds on the findings of our pilot project [16], using the pilot’s prototype as the foundation for developing the co-design activities.

This project builds on the findings of our pilot project [16], engaging stakeholders in phase 1 and advancing to the development and validation of the platform in phase 2. The pilot’s prototype serves as a foundation for developing the co-design activities. Our project is guided by the Consolidated Framework for Implementation Research, which considers 5 domains for effective intervention development and implementation: innovation, outer setting, inner setting, individuals, and implementation process [20]. The innovation domain includes the construct’s trialability and evidence base, which we addressed in our pilot work [16] and systematic review [21]. We will consider constructs in each domain that influence use of our platform by consumers including innovation, complexity and usability, consumer needs and preferences, feedback, design, and engagement [20,22-24].

### Phase 1: Engage With Stakeholders to Define Platform Development Standards

Phase 1 involves interviews, focus groups discussions, and co-design workshops with 3 specific stakeholder groups: consumers, health care professionals, and regulators (Table 1). Consumers eligible for inclusion must be 18 years of age or older, take regular medications or have previously experienced an adverse event from any medication, and own a smartphone or tablet. Health care professionals must be practicing as medical doctors, pharmacists, or nurses in Australia, and regulators will be eligible if they are involved in the postmarketing assessment of safety information for human medicinal products within Australia. Individuals will be excluded if they cannot speak, read, or write in English, or do not provide informed consent.

We will use purposeful sampling to recruit consumers with broad demographic variation to ensure that they represent consumers across different ages and social groups, levels of education and experience, and who have a range of health conditions and use a range of medications to ensure that our platform meets the needs of diverse groups of people. In addition, health care professionals and personnel who work for the medicine regulators will be recruited. All participants will be asked to participate in all 3 activities: semistructured interviews, focus group discussions, and co-design workshops. Attrition is natural in longitudinal projects such as this, and we will mitigate this by recruiting additional participants.

The interviews, focus group discussions, and co-design workshops will be conducted face-to-face where possible and audio- or video-recorded where participants agree. If participants are located interstate from the researchers or prefer to engage remotely for convenience, interviews, focus group discussions, and co-design workshops will be conducted on the web. When preferences differ, separate face-to-face and web-based workshops will be organized to accommodate participants’ preferred formats. All recordings will be transcribed verbatim, manually coded inductively, and analyzed thematically in ATLAS.ti software (ATLAS.ti Scientific Software Development GmbH) [25]. Using the Capability, Opportunity, Motivation, Behavior (COM-B) model [26], a well-established model for analyzing and modifying behaviors, the themes identified will inform the recognition of barriers and facilitators to adopting a digital platform for reporting AMEs. The themes will also be used to highlight key design features essential to meeting the needs and preferences of each of the stakeholder group. Based on these analyses, persona profiles (which will be used to summarize the identified needs, priorities, motivations, goals, and challenges of each user archetype) and user journey maps [27] (which will be used to visualize and document participants’ discussions of the likely experience personas might have during the process of reporting or reviewing AMEs as they move through the system) will be developed which will be used to guide subsequent phases of the project.

**Table 1:** Description of the different activities in phase 1 of the project.

Activity	Number	Aim	Duration	Outcome
Individual semistructured interviews	Ten to fifteen consumers; 10 health care professionals and regulators.	To understand their experience with, or perspectives toward medicine use and the current AME^a^-reporting process in Australia. To enable participants to offer their ideas and insights in confidence without the possible influence of bias from other participants.	Approximately 60 minutes per interview.	Findings will be used to develop personas that will then be discussed in focus group discussions to determine their accuracy and thoroughness as exemplar of user groups.
Focus groups discussions	Two focus-group discussions with each of the 3 stakeholder groups.	To consider and define the respective needs, priorities, and motivations the personas—developed based on the findings from the interviews—might have for reporting an AME and to define the reporting goals for the platform.	Each stakeholder focus group discussion will run for 2 hours.	Outcomes from the discussions will inform the development and delivery of the co-design workshops.
Co-design workshops	Three co-design workshops held for the same stakeholders collectively.	To employ the co-designed user personas to build a user journey map as a means to evaluate the processes of AME detection, management, and reporting, generate and agree on notional platform content and feature set to guide its subsequent development.	Each co-design workshop will last 3-4 hours and will contain multiple activities, each varying in duration but capped at 60 minutes. Regular breaks will be incorporated between activities to maintain engagement and focus.	The workshops will result in user journey maps which will be used to visualize and document participants’ discussions of the likely experience personas might have during the process of reporting or reviewing AMEs as they move through the system.

^a^AME: adverse medicine event.

### Phase 2: Develop the Platform and Interactive Data Visualization Tool

The second phase of the project focuses on the development of the platform. First, we will work with a software developer and user interaction designers to develop the underlying relational structure that received data from users might have, how the data function in terms of reporting, and what principle features a front-end user experience might have for gathering them (phase 2a—platform development). At a minimum, the project aims to achieve a fully functioning web-based reporting platform, a mobile app, or both, based on findings from phase 1. It will include a fully refined user interface and database integration and search function to operate with the TGA’s Database of Adverse Event Notifications [28]. To ensure equity across stakeholder interests, we will continue to adhere to co-design principles throughout this phase by inviting consumers, health care professionals, and regulators to participate in this process. The platform development will undergo cycles of iteration and review where stakeholders will test, compare, and contribute to decision-making on the content, form, and function of the platform through each iteration. We will perform the iterative cycles with stakeholders until no new issues are identified with the platform. We anticipate that the process will require up to 5 cycles before saturation and a deployable outcome is achieved. Stakeholders recruited in phase 1 will be invited to participate in this process, and additional participants will be invited to compensate for any attrition.

Next, as part of our communication and engagement strategy, we will develop an interactive data visualization tool or dashboard to translate and disseminate the data collected to the public, health care professionals, and regulators (phase 2b—development of interactive data visualization tool). The visualization tool will be implemented with back-end integration in the digital reporting platform and developed collaboratively through 2 co-design workshops with 8 stakeholders. The visualization tool will be configured to allow both consumers and their health care professionals to access the data they provided, obtain information on the medicines they are taking, and compare their experience with those reported by other consumers taking the same medicines. A separate visualization tool will be developed for use by regulators and will include further levels of configuration necessary for them to examine data relative to their decision-making. All consumer reports will be reviewed by study investigators (clinicians) for causality assessment (ie, likelihood that the medicine caused the observed AME) using the Naranjo probability scale [29]. The causality assessment will be done to determine whether the platform has collected all the information needed for a causality assessment.

### Phase 3: Digital Platform Implementation and Evaluation

In the third phase of the project, we will implement the new platform in general practices and pharmacies in South Australia and assess its impact for (1) increasing AME detection, (2) improving AME management, and (3) increasing AME reporting and enhancing existing TGA workflows.

**Figure 2:**
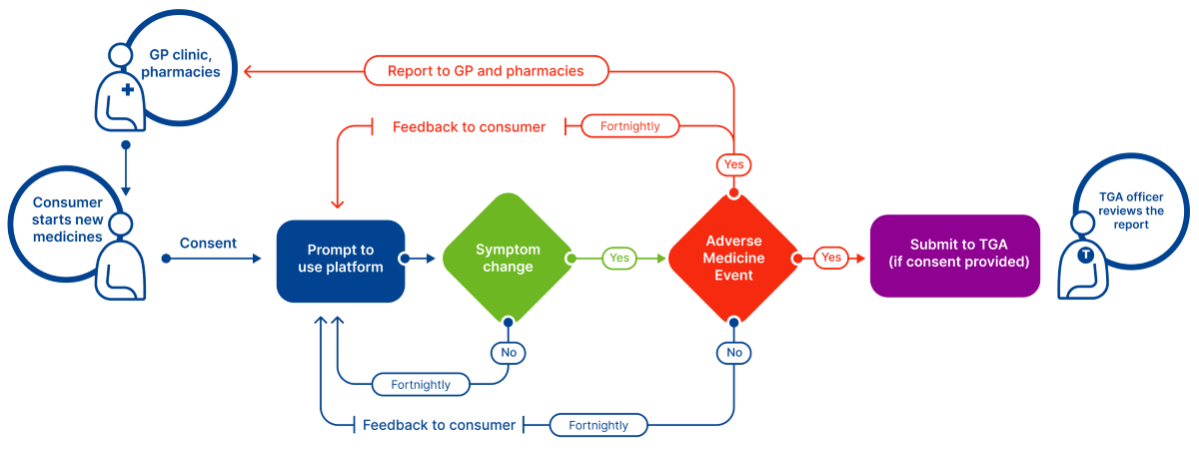
Flowchart for platform implementation in Australia. GP: general practitioner; TGA: therapeutic goods administration.

A quasi-experimental study will be conducted to involve consumers who have recently begun taking new medicines. This is because most AMEs tend to occur within 4 weeks of patients commencing new medications [30]. Depending on results from phases 1 and 2, criteria for further inclusion may be specified (eg, consumers initiating medicines with a black triangle warning). Eligible consumers will be identified initially by general practices and pharmacies applying the inclusion or exclusion criteria to their software (eg, Doctors Control Panel software [Doctors Control Panel Software Pty Ltd] [31] and dispensing software used in the pharmacies). Eligible consumers will then be approached by a dedicated research assistant via phone, text message, or email to assess their eligibility, discuss the details of the project, answer any questions, and facilitate the process of obtaining informed consent from interested individuals. We will seek consent to send information reported by consumers to their health care professionals and regulators (ie, the TGA). Consumers will then be prompted to use the platform to report whether they have experienced any suspected or possible AME. Consumers will access the platform either by downloading the mobile app or by visiting the website. Where the consumer has consented, a report will be sent electronically to their general practitioner and pharmacist to enable targeted assessment for managing the AMEs. For those who provided consent, consumer reports will also be submitted to the TGA. Subsequent prompts to use the platform will be sent to the consumers via automated text messages. The frequency of sending the text messages will be determined based on co-design workshops with stakeholder groups. Consumers will be given a summary report of their data and access to the interactive data visualization tool (Figure 2).

Based on interventions to improve AME reporting [32], which report a relative risk of 2.04 (57% in intervention group vs 28% in controls), α value (significance level: rejecting the null hypothesis when it is actually true) of .05, and power of 80%, a total of 80 consumers experiencing and reporting AME is needed. Assuming that 20% of consumers starting medicines experience an AME [33] and half of the consumers will report their AMEs with targeted prompts from the research team [34], we aim to recruit 800 people for phase 3. The sample size was calculated based on Statistical Power Analysis using R [R Foundation for Statistical Computing] [35]. Initial database analysis in one of our participating clinics with 8 full-time equivalent general practitioners indicated that each general practitioner prescribes a medicine that is new to the patient for about 40 people per month (320 per month for the practice), supporting the feasibility of recruitment of 800 participants within 6 months.

To assess whether the platform improved AME detection, interrupted time series analysis will be used to determine the proportion of AME detected before and post platform implementation in the general practices. A random sample of up to 1000 patient records in the participating general practices will be manually reviewed by nurses at the practices to determine the number of AMEs detected up to 3 months prior to platform implementation. Furthermore, we will describe the number of AME reports submitted to the TGA by our study participants post platform implementation.

Finally, we will conduct 2 focus group discussions with the project team and stakeholders to evaluate the implementation process (what works, where, and why) [20], the acceptability of, and satisfaction with the platform. Questions related to the implementation process will be adopted from the Consolidated Framework for Implementation Research interview guide tool [20]. The focus group discussions will be audio-recorded, transcribed, and analyzed using thematic analysis [25].

### Ethical Considerations

Ethics approval for phases 1 and 2 has been received from the University of South Australia human research ethics committee (application ID 204984). Ethics approval will be sought from the same committee prior to starting phase 3. Informed consent will be sought from all participants. All data obtained during the project will be deidentified. Participants in any phase of the project will receive compensation at an hourly rate, provided as either a gift voucher or a direct bank transfer in Australian dollars (Aus $). Compensation rates, approved by the human research ethics committee, are set at Aus $35 (US $21.85) per hour for consumers, Aus $200 (US $124.88) per hour for medical doctors, and Aus $100 (US $62.44) per hour for pharmacists and nurses. Furthermore, participants attending in person will receive a transportation allowance of Aus $30 (US $18.73). No compensation will be provided to regulators, as they are prohibited from accepting it.

## Results

This project is initiated in 2023 and will run until 2026. Phase 1 will result in persona profiles and user journey maps that define the standards for the user-friendly web-based communication platform and interactive data visualization tool or dashboard. This platform will be further developed and improved in phase 2. The platform will contain fully refined user interface features, an icon system for nonverbal communication, and integration with TGA’s Database of Adverse Event Notifications to support the reporting of AMEs by consumers and health care professionals. The final phase—phase 3—will provide insights of the implemented platform regarding its impact on AME detection, management, and reporting. Altogether, the efforts will result in a platform through which consumers can report AMEs to their general practitioners and pharmacists and TGA. We will publish findings progressively as we complete our analyses. In addition to the traditional research outputs (journal articles and conference papers), the designers on our project team will develop a series of nontraditional research outcomes including the dissemination of visual outcomes in the form of a public exhibition, either on the web or in person. We will organize public displays of the visual works at multiple venues in Australia to increase awareness and discussions about the importance of detecting, managing, and reporting AMEs. We will promote the exhibitions and project findings through our teams’ respective institutions’ media platforms.

## Discussion

### Expected Findings and Implications

Medicine safety is complex and requires well-developed systems, strategies, and processes to keep consumers safe. Effective systems and strategies for AME detection, management, and reporting are crucial to ensure that medicines are used safely and effectively. However, AME reporting by consumers remains low [14]. Instead, consumers were generally more likely to report AMEs to doctors or pharmacists [36], potentially stemming from inadequate or lacking systems that enable the proactive detection and management of AMEs. Our project will bring these 2 aspects together to build what we hypothesize to be a single, readily available solution that integrates AME detection and management by consumers in consultation with their general practitioners and pharmacists and which also potentially benefits AME reporting to regulators. As such, our project aims to serve both patient-level clinical needs and population-level regulatory needs on medication safety issues.

The level of end user involvement during the development phase of digital interventions that are implemented in practice is unclear. Despite the development of numerous digital interventions to improve medicine management and safety, the minimal engagement of end users in this process and a failure to meet their needs adequately result in the low adoption of these interventions in practice [37]. For instance, the implementation of GuildCare (GuildLink), an AME surveillance system designed for Australian community pharmacists, was introduced in 2014. While the initial year saw a notable increase in AME reporting rates to the TGA, the subsequent year saw a decline, hinting at challenges in maintaining sustained adoption [38]. Factors influencing the adoption and ongoing use of digital health technologies include cost, simplicity of language, ease of use, design, scientific evidence base, motivation, and perceived value by end users [22-24]. To effectively tackle the challenges related to fulfilling the requirements of stakeholders and overcoming low adoption rates, our project takes a unique approach by grounding it in a multidisciplinary ideology from its outset. This includes collaborating with experts from various disciplines including medicine safety, co-design, user-experience design, communication design, psychology, engagement, and cybersecurity to address fundamental issues that predict successful implementation of the system in practice. This partnership, grounded in a co-design methodology, also represents one of the first instances where a digital intervention for AMEs is coproduced with consumers, health care professionals, and the regulators. The approach seeks to ensure that the digital intervention directly addresses the needs of the 3 stakeholder groups, thereby increasing the likelihood of adoption in practice and ensuring its long-term sustainability.

AME reporting by consumers has the potential to improve the safety of medicines. In a previous study conducted in Australia, despite acknowledging limited awareness, consumers expressed a positive attitude toward AME reporting [15]. The perceived lack of benefits for the reporting consumer, however, was recognized as a barrier to the reporting process [15]. The significance of our proposed platform lies in its potential to incorporate consumers’ voices into their medicine and health care journey, enabling consumers to report AMEs to their health care professionals and to the regulators. If successfully implemented, the proposed platform has the potential to result in an increase in the proportion of consumer AME reports submitted to the TGA, which currently accounts for only 3.4% of the total reports submitted to the TGA [39].

There has been a decline in the percentage of AME reports from doctors in Australia, decreasing from 28% in 2003 to 4% in 2016 [36]. The potential increase in participation by consumers through use of our proposed platform may contribute to increased identification of safety signals. By streamlining the AME reporting process to the TGA, our platform has the potential to contribute to more timely detection and verification of potential medicine safety signals. This initiative addresses a national [40] and global health priority [41] and addresses 2 components of the Australia’s National Strategy for Quality Use of Medicines: monitoring outcomes and improving people’s ability to solve problems related to medicines, such as negative effects.

The introduction of a visualization tool as part of the platform has the potential to enhance end user interaction and participation in research and may facilitate early and effective communication of safety issues to relevant stakeholders. The development of interactive data visualization tools marks a creative initiative to enhance communication and transparency between consumers and regulators. While visualization tools for conveying important public health issues have become common, especially during the Covid-19 pandemic, they frequently lack transparency in describing the development process, fail to engage end users in design and development, and leave uncertainty about whether they adequately meet the needs of those end users [42]. Our user-centric co-design methodology for this project has the potential to ensure that the visualization tools effectively meet the diverse needs of consumers, health care professionals, and regulators alike.

### Limitations

First, the success of our digital reporting platform will ultimately rely on the level of user engagement and participation. Robust stakeholder engagement strategies, including co-design workshops and ongoing collaboration, do not guarantee user uptake and continued use. Factors beyond our control, such as accessibility to devices, individual motivation and preferences, and previous negative experiences with reporting, may influence the level to which consumers actively engage in this new digital platform. The introduction of any new system, service, or technology is frequently considered an additional burden or challenge when implemented in practice. However, our early engagement strategy with stakeholders, from phase 1, is designed to potentially mitigate some of this resistance and to increase the chances of adoption. Second, the reliance on interrupted time series analysis for outcome evaluation introduces potential confounding factors that may not be fully accounted. Third, the small number of consumers recruited in phase 3 means that we will not be able to determine whether the platform had an overall effect on the number of consumer reports to the TGA, or whether the reports from our platform helped generate new or different medicine safety signals. Another potential limitation is that phase 3 will be conducted in South Australia, which may restrict the generalizability of findings to the broader Australian population or internationally. However, this limitation will be mitigated by incorporating insights from participants across multiple states in Australia during phases 1 and 2. Finally, certain processes in our project rely on interim review or support from members of the research team. These workflows and processes will require revision and adaptation when implemented in clinical practice.

### Conclusions

This paper describes our co-design project that will actively involve key stakeholders in the development and evaluation of a new digital platform for AME detection, management, and reporting, with a central focus on consumers. The use of a co-design methodology ensures the incorporation of the perspectives and requirements from consumers, health care professionals, and regulators—a crucial element for fostering the adoption and sustainability of the intervention. The project harnesses the potential of both consumers and technology to address the existing challenges in underdetection and reporting of AMEs to health care professionals and regulators. The overarching goal is to enable consumers to actively participate in medication safety-related matters, thus enhancing the quality of their lives, influencing clinical decisions related to their health, and contributing to overall medicine safety.

## Abbreviations

AME: adverse medicine event
COM-B: Capability, Opportunity, Motivation, Behavior
TGA: therapeutic goods administration
